# Supplementary material for: Thirteen dubious ways to detect conserved structural RNAs
Source: IUBMB Life. Author manuscript; Available in PMC 2024 Jul 10. (PMC11234323; doi:10.1002/iub.2694)

cmf.M0557056_ext_liftover.hg38.infernal.cmalign_1

tr_1

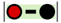

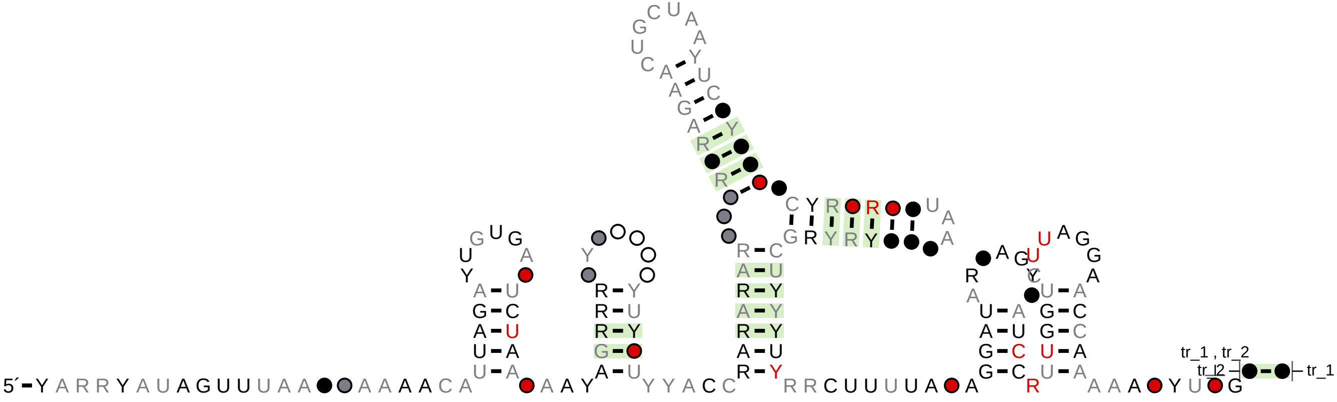

tr_2

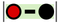

Supplement: supplementary_material [file NIHMS1907110-supplement-supplementary_material.gz › supplemental_material/Table1/CRS.structural.tar.gz!/selected/selected_noRfam/cmf.M0557056/R-scape_cma/cmf.M0557056_ext_liftover.hg38.infernal.cmalign_1.fold.R2R.sto.pdf]
